# Lower serum uric acid levels are associated with depressive symptoms in a Japanese general population: A population-based cross-sectional study

**DOI:** 10.1371/journal.pone.0311971

**Published:** 2024-12-11

**Authors:** Daiki Takekawa, Hirotaka Kinoshita, Yoshikazu Nikaido, Takashi Kudo, Tatsuya Mikami, Kazuyoshi Hirota

**Affiliations:** 1 Department of Anesthesiology, Hirosaki University Graduate School of Medicine, Hirosaki, Aomori, Japan; 2 Department of Metabolomics Innovation, Hirosaki University Graduate School of Medicine, Hirosaki, Aomori, Japan; 3 Innovation Center for Health Promotion, Hirosaki University Graduate School of Medicine, Hirosaki, Aomori, Japan; 4 Department of Anesthesiology, Aomori Prefectural Central Hospital, Aomori, Japan; Chiba Daigaku, JAPAN

## Abstract

Uric acid (UA) is a final product of purine metabolism and has neuroprotective effects. It has not been established whether serum UA levels are associated with depressive disorder. Thus, we investigated whether serum UA levels are associated with depressive symptoms in a Japanese general population. We used the Iwaki Health Promotion Project 2022 data (737 subjects) in this cross-sectional study. The Center for Epidemiologic Studies Depression Scale (CES-D) was used to assess the prevalence of depressive symptoms. Subjects with CES-D scores ≥16 were assigned to the Depression group. We compared characteristics and laboratory data (including serum UA) between the Depression and Non-depression groups and performed a multivariable logistic regression analysis to investigate whether their serum UA levels were associated with depressive symptoms, after adjusting for possible confounding factors. We analyzed the cases of 705 subjects: the Depression group (n = 142) and the Non-depression group (n = 563). The Depression group’s serum UA levels were significantly lower than those of the Non-depression group. The multivariable logistic regression analysis demonstrated that lower serum UA levels were significantly associated with the depressive symptoms. In conclusion, lower serum UA levels in this Japanese general population were significantly associated with the depressive symptoms.

## Introduction

Depressive disorder is one of the leading causes of disability [1], and the prevalence of depression has increased over the past decade [2]. Diagnosing depressive disorder is often complicated due to its various symptoms, and thus more effective strategies for achieving an early diagnosis of depressive disorder are desired.

The precise mechanisms that underlie the development of depressive disorder have not been established; however, neuroinflammation due to inflammatory cytokines released by activated microglia in the central nervous system (CNS) has been reported to play a key role in the development of depressive disorder [3,4]. Indeed, increased levels of peripheral-blood inflammatory cytokines are observed among individuals with depressive disorder, and successful treatment for depressive disorder can decrease inflammatory cytokine levels [5]. Uric acid (UA) is a final product of purine metabolism, and hyperuricemia is well known to be one of the risk factors for gout, type II diabetes mellitus, chronic kidney disease, and cardiovascular disease [6]. UA also has neuroprotective effects, since it functions as a major antioxidant [7], and antioxidants are reported to have therapeutic effects against neuroinflammation [8]. As the positive correlation between serum UA levels and cerebrospinal fluid UA levels in patients with neuroinflammatory disease due to increased blood-brain barrier permeability is reported [9], serum UA levels may reflect the level of neuroinflammation. Indeed, an animal study showed that higher serum UA induced oxidative stress and caused neuroinflammation in rats [10]. Regarding human study, hypouricemia is reported to be associated with the development of Alzheimer’s disease and that of postoperative delirium, suggesting that neuroinflammation may be involved in the pathophysiological mechanism of depressive disorder as well [11,12].

However, the published findings regarding the association between serum UA levels and depressive disorder are inconsistent. Although one investigation indicated that serum UA levels were significantly lower in patients with depressive disorder compared to healthy controls [13], multivariate analyses were not performed in that study. Another study indicated that serum UA levels were significantly higher in patients with depressive disorder compared to those without depressive disorder [14], although the sample size of that study was small. Inpatients who are aged more than 18 years with recurrent depressive episodes, major depressive disorder, depression with anxiety, or bipolar disorder depressive episodes were included in the former study [13]. On the other hand, inpatients who are male and aged 13 to 25 years with early symptoms of depression who had not received psychiatric medication were included in the latter study [14]. These patients’ background differences may have led to the difference in results.

It has thus not been established whether serum UA levels are associated with depressive disorder. We conducted the present study to examine the association between serum UA levels and depressive symptoms in a general population comprising a relatively large sample size. We speculated that if serum UA levels can be used to predict depressive symptoms, the measurement of serum UA could contribute to the diagnosis of depressive disorder.

## Materials and methods

### Study procedures and subjects

This cross-sectional study was approved by the Ethics Committee of the Hirosaki University Graduate School of Medicine (2023–110) and used the data of the Iwaki Health Promotion Project 2022 [15]. The present study was publicized on our university homepage using an opt-out approach with which subjects are included in the research unless they give their express decision to be excluded. As all subjects in the Iwaki Health Promotion Project 2022 gave written informed consent for the publication of their data in the original study, the requirement of informed consent from each subject was waived for the present study, and the Ethics Committee approved the waiver. We accessed the data on January 4^th^ 2024, and did not access to information that could identify individual participants.

The study included 737 volunteers living in the Iwaki district of the city of Hirosaki, Japan. We excluded participants with clinical diagnosis of depressive disorder including bipolar disorder (in order to avoid the effect of antidepressants to depressive symptoms), those who were under treatment with an antihyperuricemic (in order to avoid the effect of antihyperuricemic to serum UA level), and those with missing data. Demographic data and medical information were obtained from self-questionnaires and interviews. Blood samples were obtained from the medial cubital vein of the subject in the sitting position at fasting in the early morning.

We measured the subjects’ serum UA levels in addition to the white blood cell count, hemoglobin, platelet count, creatinine, aspartate transferase, alanine transferase, hemoglobin A1c, interleukin (IL)-6, tumor necrosis factor-alpha (TNF-α), and high-sensitivity C-reactive protein (hs-CRP). The Center for Epidemiologic Studies Depression Scale (CES-D) was used to assess the prevalence and severity of depressive symptoms. This scale is a short self-report scale designed to measure depressive symptomatology in a general population. The maximum score is 60, and higher scores are associated with greater depressive symptoms. We assigned the subjects with a CES-D score ≥16 to the Depression group; the subjects with CES-D scores <16 were assigned to the Non-depression group.

### Statistical analyses

Demographic data, medical information, and other variables are presented as the median (with 25th to 75th percentiles) or number and percentage of each group. The differences between the Depression and Non-depression groups were evaluated with Fisher’s exact test for categorical variables and the Mann-Whitney test for continuous variables. We performed a multivariable logistic regression analysis to investigate whether serum UA levels are associated with depressive symptoms (i.e., CES-D scores ≥16) after adjusting for possible covariates. The subjects’ serum UA level, age, and sex were forced into the model as explanatory variables. Other variables were included in the model based on existing knowledge regarding their influence. The presence of hypertension and that of diabetes mellitus were included in the model, because individuals with depressive disorder are reported to be more likely to have these diseases [16]. We also included variables with a p-value <0.1 in a univariable analysis in the model; as a result, smoking habit, hemoglobin level, and creatinine level were applied. We used the variance inflation factor (VIF) to check for multicollinearity among the variables. The results are expressed as the adjusted odds ratios (aORs) with corresponding 95% confidence intervals (CIs).

All available data was used all available data to maximize the power and generalizability of the results in the present study.

All data analyses were performed with EZR software ver. 1.27 (Saitama Medical Center, Jichi Medical University, Saitama, Japan). Probability (p)-values<0.05 were considered significant in all tests.

This manuscript adheres to the applicable TRIPOD guideline [17].

## Results

### Characteristics of subjects

**Fig 1:**
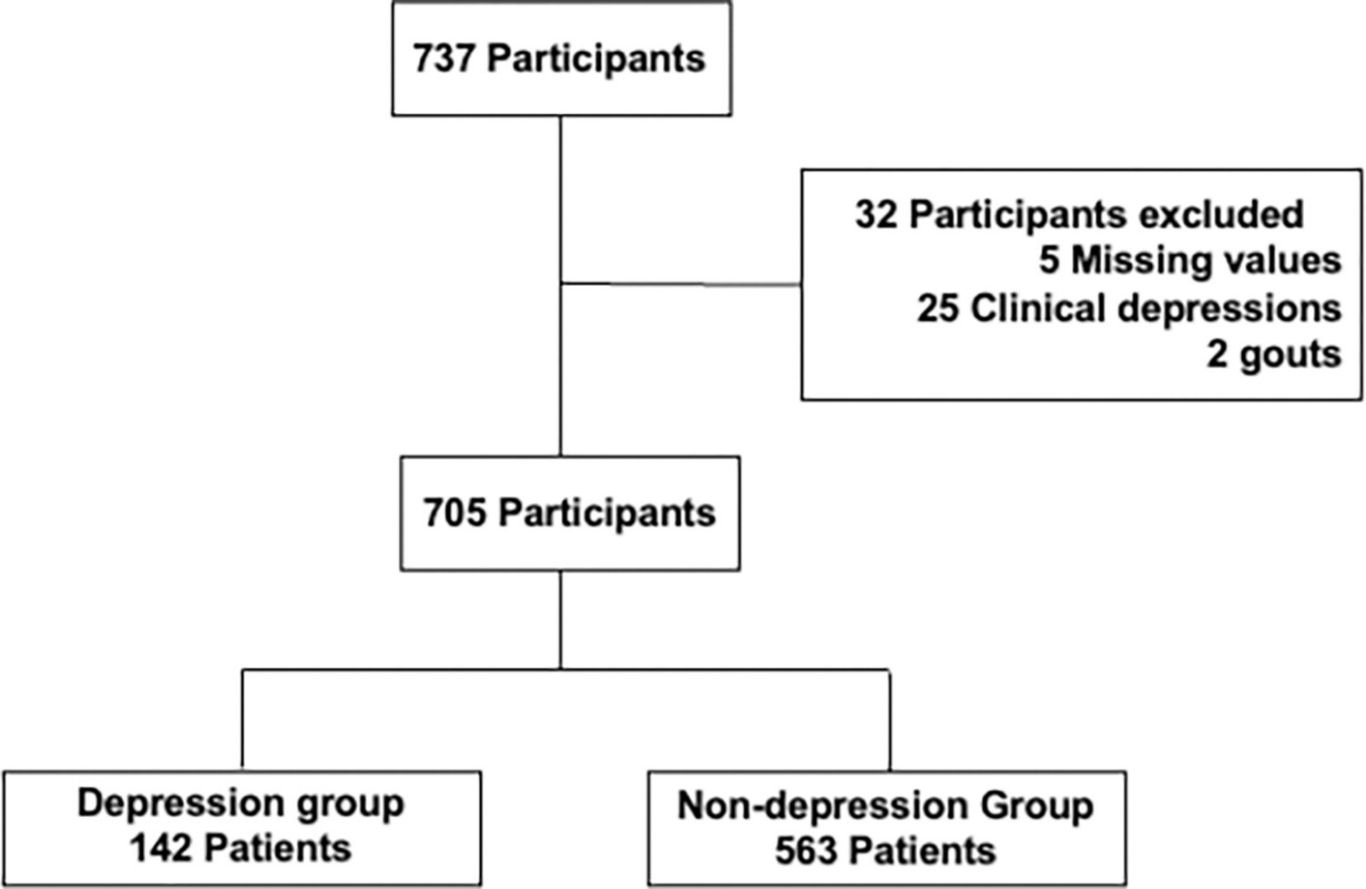
Flowchart outlining patient selection and grouping process.

Of the 737 subjects, the cases of a final total of 705 subjects were analyzed (Fig 1). Of the 705 subjects, 142 subjects comprised the Depression group, and 563 subjects comprised the Non-depression group. The subjects’ characteristics are summarized in Table 1. The hemoglobin and serum creatinine levels were significantly lower in the Depression group. The rate of a smoking habit was significantly different between the groups. Of course, the CES-D scores were significantly higher in the Depression group. The data of other variables were not significantly different between the subject groups.

**Table 1:** Characteristics of the Depression and Non-depression groups.

	Depression	Non-Depression	P value
N	142	563	
Male, n	49 (34.5%)	244 (43.3%)	0.057
Age, yrs	50.0 (37.0, 65.8)	55.0 (42.0, 66.0)	0.104
BMI, kg/m^2^	22.6 (20.4, 24.5)	22.7 (20.8, 25.3)	0.112
Medical history			
Hypertension, n	41 (28.9%)	126 (25.9%)	0.523
DM, n	9 (6.3%)	49 (8.7%)	0.494
Dyslipidemia, n	24 (16.9%)	104 (18.5%)	0.716
Stroke, n	1 (0.7%)	6 (1.1%)	1.000
CAD, n	2 (1.4%)	10 (1.8%)	1.000
Smoking			<0.011*
Current, n	22 (15.5%)	92 (16.3%)	
Past, n	32 (22.5%)	195 (34.6%)	
Never, n	88 (62.0%)	276 (49.0%)	
Alcohol consumption			0.379
Current, n	66 (46.5%)	297 (52.8%)	
Past, n	9 (6.3%)	28 (5.0%)	
Never, n	67 (47.2%)	238 (42.3%)	
CES-D score	20.0 (17.0, 24.0)	7.0(3.0, 11.0)	
Laboratory data			
WBC, /10^3^	5.1 (4.3, 6.3)	5.1 (4.2, 6.0)	0.387
Hemoglobin, g/dL	13.8 (13.0, 14.6)	14.1 (13.2, 15.1)	0.040*
Platelet, /10^4^	24.1 (21.6, 29.1)	24.2 (20.6, 28.2)	0.414
AST, U/L	20.0 (18.0, 27.0)	21.0 (18.0, 25.0)	0.936
ALT, U/L	18.0 (12.0, 25.0)	18.0 (13.0, 25.0)	0.444
Creatinine, mg/dL	0.69 (0.60, 0.83)	0.73 (0.63, 0.86)	0.027*
HbA1c, %	5.6 (5.3, 5.8)	5.5 (5.4, 5.8)	0.764

Differences between the Depression and Non-depression groups were examined by Fisher’s exact test for categorical variables and the Mann-Whitney test for continuous variables. Data are n (percentage of each group) or median (25th to 75th percentiles). ALT: Alanine transferase, AST: Aspartate transferase, BMI: Body mass index, BW: Body weight, CAD: Coronary artery disease, CES-D: Center for Epidemiologic Studies Depression Scale, DM: Diabetes mellitus, HbA1c: Hemoglobin A1c, WBC: White blood cell

*p<0.05.

### Uric acid and inflammatory markers

Table 2 provides the details of the serum UA level and inflammatory marker levels of each group. The serum UA levels of the subjects in the Depression group were significantly lower than those of the Non-depression group (4.75 mg/dL vs. 5.0 mg/dL respectively, p = 0.004). There were no significant between-group differences in the IL-6, TNF-α, or hs-CRP levels.

**Table 2:** Serum UA and inflammatory markers.

	Depression	Non-Depression	P value
UA, mg/dL	4.75 (4.00, 5.47)	5.00 (4.20, 6.00)	0.004*
IL-6, pg/mL	1.12 (0.83, 1.87)	1.14 (0.83, 1.72)	0.977
TNF-α, pg/mL	5.14 (4.17, 6.23)	5.18 (4.19, 6.26)	0.955
Hs-CRP, mg/dL	0.02 (0.01, 0.06)	0.03 (0.01, 0.07)	0.213

Differences between the Depression and Non-depression groups were examined by the Mann-Whitney test. Data are median (25th to 75th percentile). Hs-CRP: High-sensitivity C-reactive protein, IL-6: Interleukin-6, TNF-α: Tumor necrosis factor-alpha, UA: Uric acid

*p<0.05.

### The association between serum UA and depressive symptoms

The results of multivariable logistic regression analyses to identify whether serum UA can be used predict the depressive symptoms after the adjustment for possible confounders are shown in Table 3. A lower serum UA level (aOR: 0.816, 95%CI: 0.673–0.988, p = 0.038) was significantly associated with the depressive symptoms. The presence of hypertension (aOR: 1.740, 95%CI: 1.050–2.870, p = 0.030) and a past smoking history (reference: non-smoker, aOR: 0.504, 95%CI: 0.361–0.944, p = 0.028) were also significantly associated with the depressive symptoms.

**Table 3:** Multivariable logistic regression analysis to identify whether serum uric acid can predict depressive symptoms.

	aOR	95%CI	P value
UA, per 1-mg/dL increase	0.816	0.673, 0.988	0.038*
Age, per 1-yr increase	0.987	0.973, 1.000	0.077
Male	1.190	0.609, 2.330	0.610
Hypertension	1.740	1.050, 2.870	0.030*
DM	0.728	0.333, 1.590	0.428
Smoking			
Never	Ref.		
Current	0.888	0.499, 1.580	0.688
Past	0.507	0.361, 0.944	0.028*
Hemoglobin, 1-g/dL increase	1.010	0.855, 1.190	0.923
Creatinine, 1-mg/dL increase	0.623	0.124,3.140	0.566

No variance inflation factor value reached 10, indicating that there was no collinearity in the model. The area under the curve was 0.623 (95%CI: 0.573–0.674). DM: Diabetes mellitus, UA: Uric acid, aOR: Adjusted odds ratio

*p<0.05.

## Discussion

We evaluated the association between serum UA levels and the depressive symptoms, and the results of our analyses demonstrated that after adjustment for possible confounding factors, lower serum UA levels were significantly associated with the depressive symptoms in a general Japanese population. The presence of hypertension and smoking history were also significantly associated with the depressive symptoms in the present population.

Neuroinflammation is reported to play an important role in the development of depressive disorder. Infection or CNS damage can activate microglia, and activated microglia release IL-6, IL-1β, and TNF-α [18]. Neuroinflammation inhibits neurogenesis in the hippocampus, which may be involved in the pathogenesis of the development of depressive disorder [19]. Indeed, brain inflammatory markers of a chronic mild-stress model of depression in mice were reported to be increased [20], and peripheral blood inflammatory markers of patients with depressive disorder were also reported to increase [5]. On the other hand, our present analyses revealed that there were no significant differences in IL-6, TNF-α, or hs-CRP between the groups of individuals classified as having or not having depression. The discrepancy in results among studies may be due to the following: (*i*) our present subjects were healthy volunteers, and (*ii*) we excluded patients with a clinical diagnosis of depressive disorder to avoid the effect of the antidepressants to the results. The neuroinflammation in such subjects may be weaker than that in patients with a clinical diagnosis of depressive disorder. We observed that peripheral-blood inflammatory markers in healthy volunteers who had depressive symptoms that were diagnosed in the same manner as that used in the present study were not increased [21].

To the best of our knowledge, the present study is the first to reveal an association between serum UA levels and depressive symptoms in a Japanese general population. The present results are consistent with a previous study that demonstrated that serum UA levels were significantly lower in patients with depressive disorder compared to healthy controls [13]. However, as that study did not perform multivariable analyses, the results did not adjust for confounder, such as comorbidities and lifestyle habit. On the other hand, the strength of this study is that the sample size was enough large to perform multivariable analyses. Thus, the present study strengthened the evidence about the association between serum UA levels and depressive symptom. In a mouse model of multiple sclerosis, an intraperitoneal administration of UA inhibited neuroinflammation, blood-brain-barrier permeability changes, and CNS tissue damage [22]. It was also reported that UA protects dopaminergic neurons against neuroinflammation by modulating the UA transporter-mediated intracellular UA level [23]. Our present analyses demonstrated that lower serum UA levels were associated with the depression. However, in our analyses, as we did not measure brain inflammatory markers, we could not directly evaluate the treatment effect of UA on neuroinflammation. Additional studies that measure cerebrospinal-fluid inflammatory markers in patients with depressive disorder are necessary to test our findings.

Our analyses also revealed that the presence of hypertension was significantly associated with increased risk of the depressive symptoms and that a past history of smoking was significantly associated with decreased risk of the depressive symptoms. Regarding hypertension, individuals with depressive disorder tend to have hypertension compared to those without depressive disorder, as the sympathetic nervous tone is likely to be increased in persons with depressive disorder [24]. Regarding a history of smoking, a cohort study using data from a large, randomized clinical trial showed that smoking cessation was associated with an improvement of mental health disorders including depression [25]. However, as our results showed that past smoking history lowered the risk of depressive symptoms compared to no smoking history, further studies to investigate the association between the smoking history and depressive symptoms are needed.

The present study has some limitations to consider. First, this was an observational study with a relatively small sample size, and there may have been undetected confounding factors that affected the results. Second, since this was a cross-sectional study, no conclusions can be made regarding causal associations between depression and possible confounding factors. Third, since this investigation was limited to Japanese subjects, possible ethnic differences were not considered. Fourth, although we measured not only serum UA but also inflammatory markers, we didn’t measure antioxidation/oxidation markers. Thus, the results were insufficient to clarify the antioxidant effect of UA. Fifth, we didn’t measure substances involved in purine metabolism such as adenine, guanine, hypoxanthine, xanthine, inosine monophosphate, 5-phosphoribosyl pyrophosphate, glycine, glutamine, and aspartate. To the best of our knowledge, there is a study that showed the relationship between purine metabolism and major depressive disorder in children and adolescents [26]. Further study to focus on the relationship between purine metabolism and depressive symptoms in general population are needed to strengthen the evidence of this study.

## Conclusions

In conclusion, after adjustment for possible confounding factors, lower serum UA levels in a Japanese general population were significantly associated with the depressive symptoms. This finding will be useful for the early diagnosis of depressive disorder in the future. Studies with greater numbers of subjects are necessary to test our findings.
